# Overexpression of Hypo-Phosphorylated IκBβ at Ser313 Protects the Heart against Sepsis

**DOI:** 10.1371/journal.pone.0160860

**Published:** 2016-08-10

**Authors:** Guang-Qing Wang, Tao Tang, Zhong-Shan Wang, Ying-Ying Liu, Li Wang, Peng-Fei Luo, Zhao-Fan Xia

**Affiliations:** 1 Department of Burn Surgery, Changhai Hospital, Second Military Medical University, Shanghai, China; 2 Department of Surgery, 91528 Hospital of PLA, Shanghai, China; Indian Institute of Science Education and Research, INDIA

## Abstract

IκBβis an inhibitor of nuclear factor kappa B(NF-κB) and participates in the cardiac response to sepsis. However, the role of the hypo-phosphorylated form of IκBβ at Ser313, which can be detected during sepsis, is unknown. Here, we examined the effects of IκBβ with a mutation at Ser313→Ala313 on cardiac damage induced by sepsis. Transgenic (Tg) mice were generated to overexpress IκBβ, in which Ser-313 is replaced with alanine ubiquitously, in order to mimic the hypo-phosphorylated form of IκBβ. Survival analysis showed that Tg mice exhibited decreased inflammatory cytokine levels and decreased rates of mortality in comparison to wild type (WT) mice, after sepsis in a cecal-ligation and puncture model (CLP). Compared to WT septic mice, sepsis in Tg mice resulted in improved cardiac functions, lower levels of troponin I and decreased rates of cardiomyocyte apoptosis, compared to WT mice. The increased formation of autophagicvacuoles detected with electron microscopy demonstrated the enhancement of cardiac autophagy. This phenomenon was further confirmed by the differential expression of genes related to autophagy, such as LC3, Atg5, Beclin-1, and p62. The increased expression of Cathepsin L(Ctsl), a specific marker for mitochondrial stress response, may be associated with the beneficial effects of the hypo-phosphorylated form of IκBβ. Our observations suggest that the hypo-phosphorylated form of IκBβ at Ser313 is beneficial to the heart in sepsis through inhibition of apoptosisand enhancement of autophagy in mutated IκBβ transgenic mice.

## Introduction

High mortality in septic patients is associated with cardiac dysfunction. Clinical studies revealed impaired systolic and diastolic dysfunction [1,2]. However, the pathological mechanism for this complication is not clear. Previous work revealed that the activation of NF-κB was one of the key signal transduction pathways in cardiomyocyte injury induced by LPS [3].

NF-κB transcription factors are homo- and heterodimers of multiple subunits including p65 (Rel A), Rel B, c-Rel, p50/p105 (NF-κB1), and p52/p100 (NF-κB2). The different combinations of subunits have unique propensities for regulation of gene expression in both physiological and pathological conditions. NF-κB proteins are constitutively retained in the cytoplasm by combination with inhibitor of kappa B proteins (IκBs). Once IκBs are degraded, NF-κB is activated and translocated from the cytoplasm to the nucleus, in which it binds to the promoter of specific genes as a transcription factor to activate or inhibit gene expression. The termination of NF-κB activity is dependent upon re-synthesis and translocation of IκBs into the nucleus. These classical functions are mainly executed by IκBα, IκBβ, and IκBε. Among them, IκBβ has many unique roles in endotoxemia [4]. Structural and functional analyses revealed that human IκBβ is phosphorylated at two serine (S313, S315) phosphorylation sites in the resting cell. When stimulated by lipopolysaccharide(LPS), the IκBβ in cardiomyocytes and macrophages is slowly degraded to allow translocation of NF-κB dimers containing p65 and/or c-Rel subunits into the nucleus through their nuclear localization signal (NLS) activities. However, the newly synthesized IκBβ is in a hypo-phosphorylated form. The hypo-phosphorylated IκBβ can expose the NLS of p65 and strengthen its binding to DNA, which may facilitate the accumulation of NF-κB−IκBβ complexes in the nucleus and prolong the activation of NF-κB. In a mitochondrial stress model, Biswas G *et al*. demonstrated that calcineurin de-phosphorylates IκBβ at Ser313/Ser315 and releases c-Rel/p50 heterodimers for translocation into the nucleus [5,6]. We also observed the presence of hypo-phosphorylated IκBβ in the heart after sepsis.

Even though de-phosphorylation of Ser-313 and Ser-315 of human IκBβ is associated with the activities of p65/p50, c-Rel/p50 and p65/c-Rel dimers; we still do not fully understand its pathological significance in sepsis. Here, we report that over-expression of mouse IκBβ mutated at Ser-313 ameliorates cardiac function in sepsis.

## Materials and Methods

### Experimental animals

Generation of mutated IκBβ transgenic mice: A full-length mouse cDNA was mutated to encode IκBβ protein in which Serine313 was replaced with alanine to prevent IκBβfrom Serine313 phosphorylation by casein kinase II (CKII) [7]. The mouse mutated IκBβ was cloned into vector pRP.Ex3d-EF1A>IκBβ*>IRES/EGFP with Gateway Technology. The target transgenic cassette was injected into FVB mouse embryos and implanted into female mice. The Tg mice were identified using genomic DNA. Three independent founder lines were identified and raised in the laboratory (Experimental Animal Center, SMMU, Shanghai, China).

Thisstudy was carried out on 6–8 week-old male FVB mice (Tg and WT, 20–25g). They were housed in individually ventilated cages lined with an absorbent beddingmaterial with no more than 6 mice per cage. The room temperature and humidity was maintained at 23±3°C and 55±10%. All animals had free access to a standard dietand water. The feeding boxes were cleaned and disinfected every 3 days, and the water was changed on a daily basis to prevent infectious diseases. Animals were inspected for signs of illness and unusual behaviour by research staff at leastonce per day. All surgeries were performed under Sodium Pentobarbital anesthesia and echocardiography was performed under inhalation anesthesia of isoflurane, and buprenorphine was administered 6 hours and 18 hours after surgery to reduce postoperative pain. For all animal experiments survival was monitored at least three times daily, and moribund animals indicated bylethargy, loss of ability to ambulate (inability to access food or water), hunched posture or rough hair coatwere sacrificed by cervical dislocation under intraperitoneal injectionof sodium pentobarbitone (50 mg/kg). There were no unexpected animal deaths. We used the humane endpoints as described above to euthanize the animals in order to minimize suffering and distress.

All the animals were treated in accordance with the ARRIVE guidelines developed by the National Center for the Replacement, Refinement, and Reduction of Animals in Research and all animal protocols were approved by The Standing Committees on Animals of Second Military Medical University(Shanghai, China).

### Material and reagents

The reagents for Gateway Technology were purchasedfromInvitrogen(Carlsbad, CA, USA). Antibodies and cell culture reagents were as follows: Antibody for phosphoserine(Ser-313) of IκBβ was generated by immunization of rabbit with peptide LSPCS(pS)SGSDS(Abmart, Shanghai, China); the rabbit polyclonal antibody LC3, Cathepsin L and Atg5 (Abcam, Cambridge, UK); the rabbit monoclonal antibody p62, Beclin-1, GAPDH and b-actin (Cell Signaling Technology, Danvers, MA, USA); DMEM, fetal bovine serum (FBS) (Invitrogen, Carlsbad, CA, USA). Subcellular Protein Fractionation Kit for Tissues was purchased fromThermoScientific (Rockford, IL, USA). In Situ Cell Death Detection Kit, TMR red waspurchased fromRoche (Basel, Switzerland). Trizol Reagentwas purchased fromInvitrogen (Carlsbad, CA, USA). ELISA kits of IL-6, TNF-α and cTnI were obtained from R&D Systems(Minneapolis, MN, USA). All other biochemicals used were of the highest purity available and were obtained from regular commercial sources.

### Mouse cecal-ligation and puncture (CLP) model

Sepsis was induced by CLP as previously described with minor modifications [8]. Briefly, all FVB mice were kept in pathogen-free conditions. Food was removed for 16 hours before experiment. Six to eight week-old WT/Tg mice were anesthetized by intraperitoneal injection of Sodium Pentobarbital (50 mg/kg). Under sterile conditions, a 0.8-cm midline laparotomy was made to expose cecum in the lower left abdomen. The distal portion of the cecum was ligated with a 4–0 silk suture and punctured thoroughly with a 20-gauge needle twice, and then a small amount of stool was squeezed out through the punctures. After the cecum was replaced in the peritoneal cavity, the peritoneal wall and skin incisions were closed. Sham operated mice were subjected to similar laparotomy without CLP. After surgery all animals immediately received a subcutaneous injection of 1 ml of sterile saline (0.9% NaCl) for resuscitation and had free access to food and water.

### Survival assays

Tg mice and WT mice were age- and sex-matched before performing the CLP operation(n = 20). We measured the survival rates of Tg and WT mice after CLP every 8h for 3 days. The survival rates were observed and compared by log-rank test. We used humane endpoints and euthanized animals prior to their death in the survival assays as described in experimental animals.

### Blood and plasma measurement

Serum IL-6, TNF-α, and cTnI were detected with an ELISA method using a commercial kit.

### Assessment of cardiac function in vivo

Cardiac function was assessed in mice by echocardiographyin vivo as reported previously [9]. At 12 hours after CLP, anesthesia was inducedwith 3% isoflurane and maintained at 0.5 to 0.7% for the duration of the procedure. The heart rate was obtained from ECG tracing and the temperature was monitored with a rectal thermometer. Two-dimensional and M-mode echocardiography images were recorded using a Aixplorer imaging system(SuperSonicImagine). Percentage ejection fraction (EF) andfractional shortening (FS) were calculated from the M-modemeasurements in the parasternal short axis view at the level of thepapillary muscles.

### Transmission electron microscopy analysis

The heart was perfused with 0.5% glutaric dialdehyde in 0.1 M cacodylate buffer (perfusion fluid) at the speed of 3ml/min for 5 min to fix the muscle in situ. Then the freshly isolated cardiac tissue from mice was cut into small samples and immediately immersion-fixed with perfusion fluid for 3 hours. After washing with 0.1 M cacodylate buffer and 15% sucrose buffer, the samples were cut into thin sections (90 nm), which were viewed at 120 kV with a H7650 transmission electron microscope (HITACHI, Tokyo, Japan). Micrographs were taken in the Philips CM12 (10–15 per sample) by random sampling.

### Western blot analysis

50μg of cellular extracts from heart tissues were electrophoresed on a 10% SDS-PAGE gel and electroblotted onto polyvinylidene-difluoride membranes which were blocked and incubated with the antibodies mentioned previously. Immunoactive protein bands were revealed by ECL chemiluminescence reagents (ThermoScientific, Rockford, IL, USA). Densitometry was performed using a gel documentation system.

### TUNEL analysis

Myocardiac tissue was harvested from WT/Tg mice with or without CLP. Apoptotic cells in the cardiac tissue were determined by TUNEL (terminal dUTP nick-end labeling) with In Situ Cell Death Detection Kit, TMR redmentioned previously. Samples were analyzed under a microscope (Olympus, Tokyo, Japan) by an investigator blinded to sample identity.

### Flow cytometry analysisfor apoptosis

Primary cardiac cells were prepared from WT and Tg mice according to previous protocol with modification [10]. Briefly, cardiac tissue from WT and Tg mice was digested with 0.1% DispaseII (Worthington, Lakewood, USA) at 4°C over night and then 0.125% Trypsin (Gibco, Grand Island, NY, USA) at 37°C for 10 minutes. The primary cardiac cells were cultured in DMEM (Invitrogen, Carlsbad, CA, USA) containing 10% fetal bovine serum (Invitrogen, Carlsbad, CA, USA) at 37°C for 24 hours. After treatment with LPS, the primary cardiac cells were stained with both propidiumiodide (PI) and annexin V (AV)-FITC and loaded on a flow cytometer (FACSCalibur, Becton Dickinson, Franklin Lakes, USA). Data from 10,000 cells/sample were collected, and the quadrants were set according to the population of viable, unstained cells in untreated samples.

### RNA isolation and real time PCR

For isolation RNA, the cardiac tissue was homogenized in Trizol and mixed with chloroform, centrifuged at 12,000rpm for 10 min, then the upper aqueous phase was mixed with isopropanol and centrifuged at 12,000rpm for 10 min, then the total RNA was dissolved in ddH2O. cDNA was generated using OligodT as primers. Expression level of different genes was assessed with quanti-tative PCR using the StepOnePlus Real-Time PCR system (Applied Biosystems, Life Technologies, USA),and the primers (Sangon Biotech, Shanghai, China) were as follows:

TNF-α: forward 5’-TGTCTCAGCCTCTTCTCATT-3’,

reverse 5’-AGATGATCTGAGTGTGAGGG-3’;

IL-6: forward 5’-ATGAAGTTCCTCTCTGCAAGAGACT-3’,

reverse 5’ -CACTAGGTTTGCCCAGTAGATCTC-3’.

IκB-β: forward 5’-GCGGATGCCGATGAATGGT-3’,

reverse 5’-TGACGTAGCCAAAGACTAAGGG-3’

CathepsinL: forward 5’-ATCAAACCTTTAGTGCAGAGTGG-3’,

reverse 5’-CTGTATTCCCCGTTGTGTAGC-3’

GAPDH: forward 5’- AGGTCGGTGTGAACGGATTTG-3’,

reverse 5’-TGTAGACCATGTAGTTGAGGTCA -3’

EGFP: forward 5’-CAACTACAACAGCCACAACG-3’,

reverse 5’-GGTCACGAACTCCAGCAG-3’

Values were normalized to expression levels.

### Statistical analysis

Values were presented as mean ± SEM from at least three separate experiments. Data were Analyzed by one-way analysis of variance (ANOVA) and Bonferroni’s multiple comparison tests and t testusing SPSS 16.0 software. The survival rates were observed and compared out to 72 hours by log-rank test. Differences between groups were considered to be significant at *p*< 0.05.

## Results

### The response of IκBβ to sepsis in WT mouse hearts

**Fig 1:**
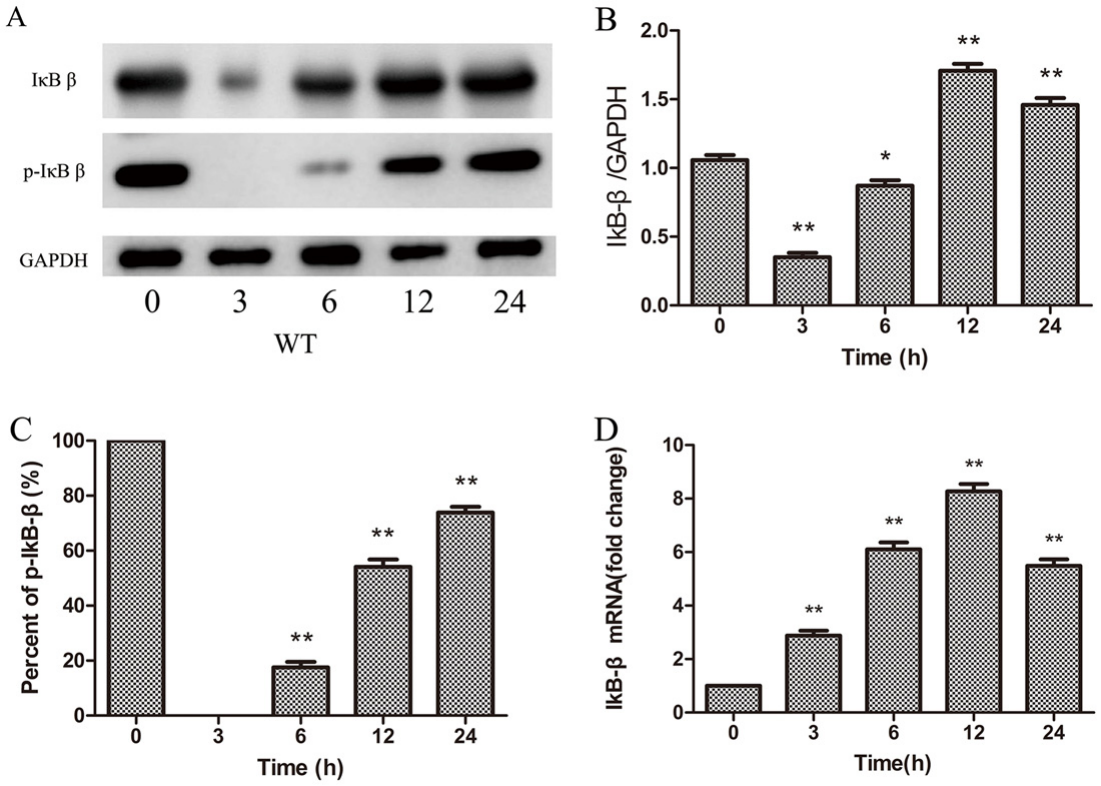
IκBβ expression in the heart from WT mice post CLP. (A) The content of IκBβ protein weredetected with Western Blotting (n = 3). (B)IκBβ Relative protein levels were quantified and normalized to GADPH (n = 3).(C) The ratio of phosphorylated IκBβ at Ser313 to the total IκBβ protein (n = 3).(D) mRNA levels of IκBβ measured by real-time PCR (n = 3). Figures are representative of three independent experiments. Values were presented as mean ± SEM,*p< 0.05, **p< 0.01,one-way analysis of variance (ANOVA) and Bonferroni’s multiple comparison tests.

Once phosphorylated by IκB kinase at Ser-19 and Ser-23 [11], IκBβ is subsequently degraded via the ubiquitin-proteasome. The IκBβ protein in the heart challenged with CLP is significantly diminished at 3h, and gradually increased at 6h, 12h, and 24h (*p*< 0.05; Fig 1A and 1B). However, the mRNA level of IκBβ steadily increases and reaches maximal levels at 12h after sepsis (*p*< 0.01; Fig 1D). Besides the change of IκBβ quantity, the phosphorylation state of mouse IκBβ at Ser-313 is also different in the heart after CLP. We detected the phosphorylation state of IκBβ at Ser-313 with western blot, there are less phosphorylated forms of IκBβ at the Ser-313 site in the newly synthesized IκBβ at 6h (17.5%),12h (54.1%), and 24h (73.9%) (*p*< 0.01; Fig 1A and 1C).

### IκBβ*-Tg mice exhibit decreased mortality and inflammatory cytokines post CLP

**Fig 2:**
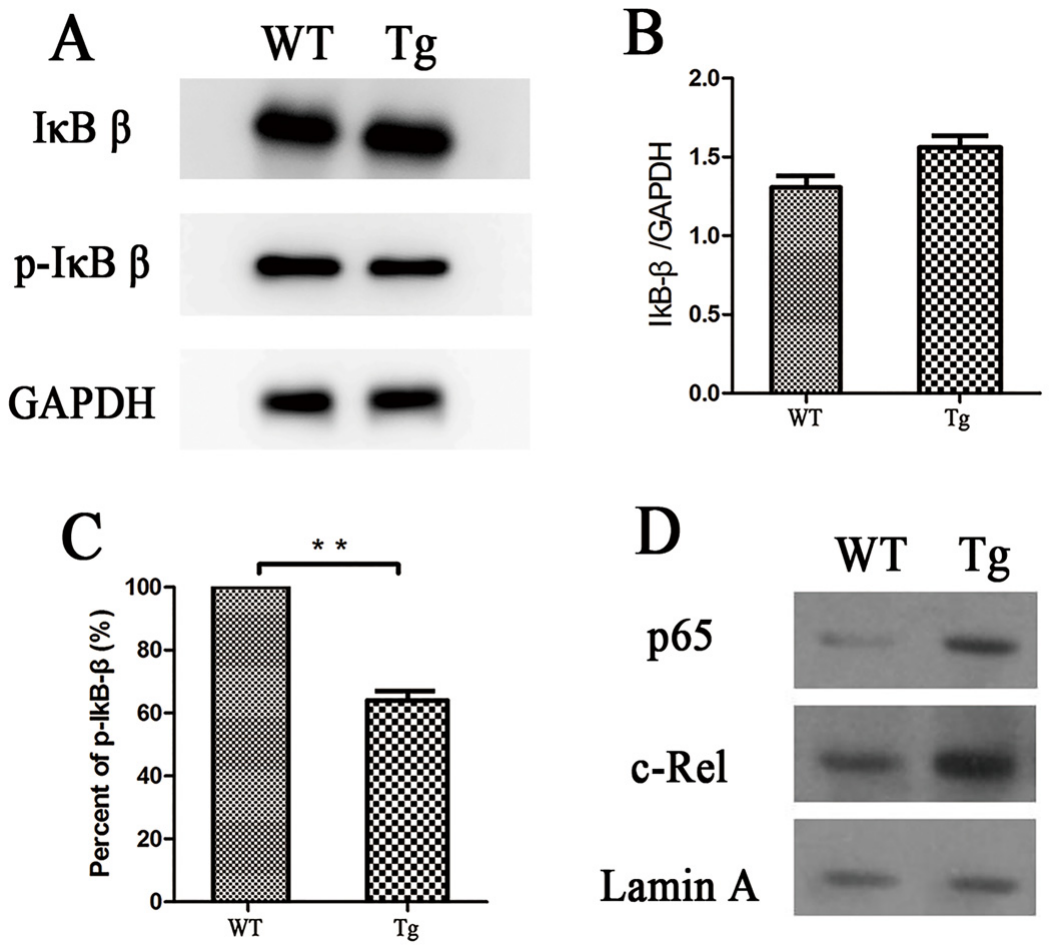
Phenotypeof IκBβand spatial alteration of NF-κB in WT and Tg mice. (A) The content of IκBβ protein were detected with Western Blotting (n = 3). (B) IκBβ Relative protein levels were quantified and normalized to GADPH (n = 3). (C) The ratio of phosphorylated IκBβ at Ser313 to the total IκBβ protein (n = 3). (D) The content of p65 and c-Rel in the nucleus were detected with Western Blotting (n = 3). Figures are representative of three independent experiments. Values were presented as mean ± SEM, *p< 0.05, **p< 0.01,t test.

**Fig 3:**
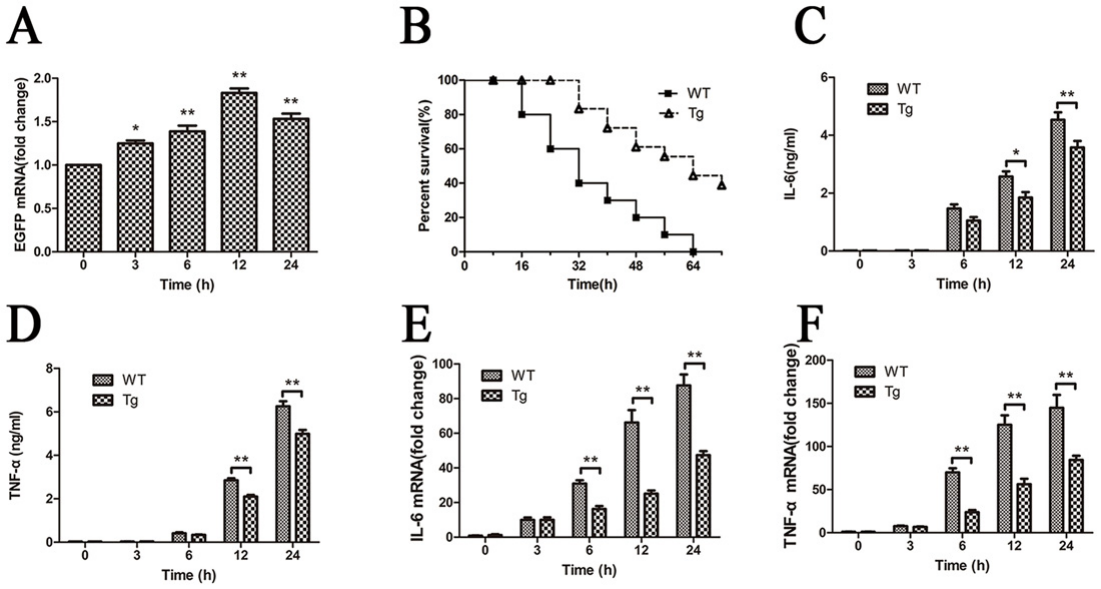
Hypo-phosphorylated IκBβ at S313 improves survival and decreases inflammatory cytokines in CLP sepsis. (A)The expression of EGFP in Tg mice post CLP. mRNA level of EGFP was examined by real-time PCR (n = 3). (B) Tg mice overexpressing hypo-phosphorylated IκBβ at Ser-313 and WT mice were age- and sex-matched before performing the CLP operation(n = 20). The survival rates were observed and compared out to 72 hours by log-rank test (*p*< 0.01). (C-D)Serum IL-6 and TNF-α levels (ng/mL) at different time-points afterCLP were detected by ELISA (n = 6).(E-F) mRNA levels of IL-6 and TNF-αwere examined by real-time PCR (n = 6). Figures are representative of three independent experiments. Values were presented as mean ± SEM,*p< 0.05, **p< 0.01,one-way analysis of variance (ANOVA) and Bonferroni’s multiple comparison tests, log-rank test.

Tg mice overexpressing IκBβ in which Ser-313 is replaced with alanine ubiquitously (simulating the IκBβ-hypophosphorylated form at Ser-313), termed “IκBβ*”, were generated as described in Methods. The phenotype data of Tg mice were shown in Fig 2A–2C. The protein level of IκBβ in the heart from Tg mice was not changed significantly (*p*> 0.05; Fig 2A and 2B), while the protein level of phospho-IκBβ Ser-313 in the heart from Tg mice was significantly lower than that from WT mice (Tg 64.04% *vs*. WT 100%) (*p*< 0.01; Fig 2A and 2C). The Tg mice show a different spatial alteration of NF-κB (Fig 2D). There is a increased portion of NF-κB protein (p65, c-Rel) in the nucleus from the heart of Tg mice.Tg mice also expressed EGFP consistently (*p*< 0.05; Fig 3A) in the heart post sepsis.

The Tg mice were healthy without any detectable pathological symptoms during 5 months observation. To explore whether IκBβ* is detrimental or beneficial to the mice in sepsis, we measured the survival rates of Tg and WT mice after CLP every 8h for 3 days. WT and Tg mice had a survival rate of 60% and 100% at 24 hours, respectively. Whereas all the WT mice died at 64 hours, the Tg mice had a survival rate of 50% at this time point. Overall, the septic Tg mice had significantly increased survival after CLP compared with WT mice(*p*<0.01; Fig 3B).

The serum levels of IL-6 and TNF-α were significantly different between Tg and WT mice (*p*< 0.05; Fig 3C and 3D). Furthermore, the gene expression of IL-6 andTNF-α in cardiac tissue from Tg mice was significantly diminished compared to that from WT mice at 6h,12h, and 24h(*p*< 0.01; Fig 3E and 3F).

### IκBβ* ameliorates heart injury and decreases cardiac apoptosis

**Fig 4:**
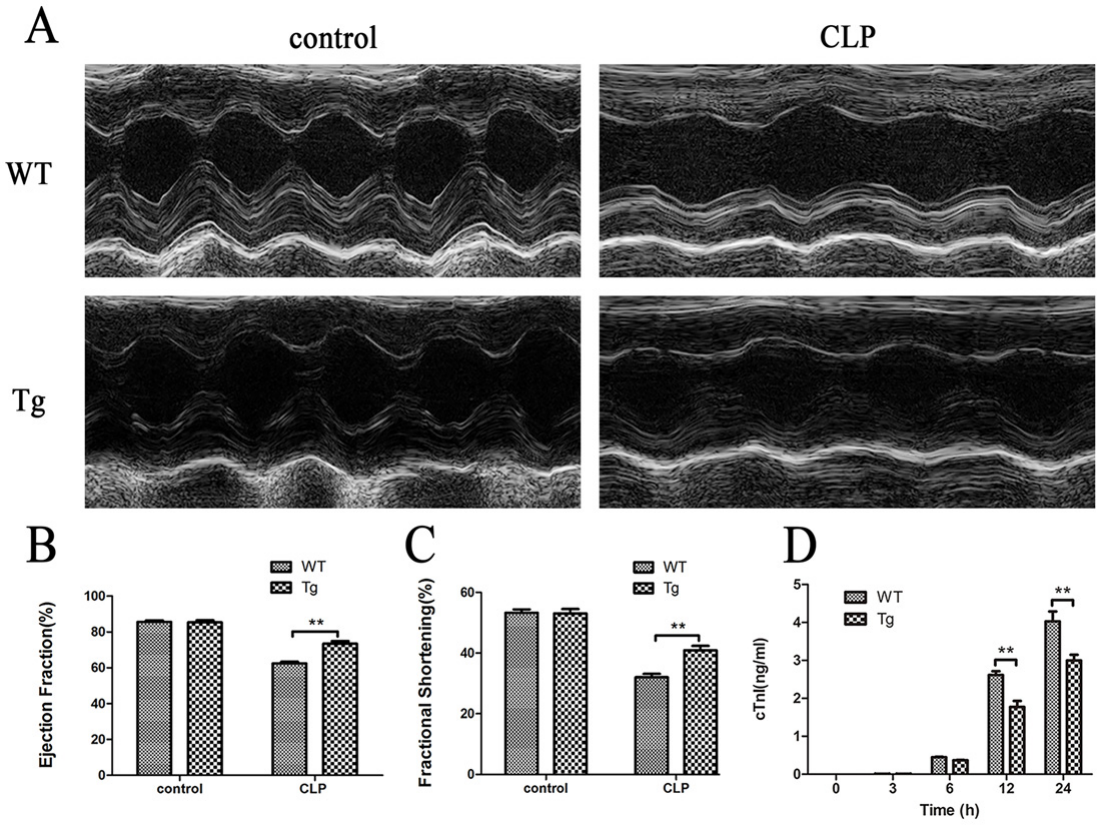
Cardiac function in WT and Tg mice. (A-C) Cardiac function was examined with echocardiography at 12 hours after CLP (n = 6). (A)RepresentativeM-mode echocardiograms. (B)ejection fraction (EF). (C) fractional shortening (FS). (D)Serum cTnI levels (pg/mL) at different time-points post CLP were detected by ELISA (n = 6). Figures are representative of three independent experiments. Values were presented as mean ± SEM,*p< 0.05, **p< 0.01,one-way analysis of variance (ANOVA) and Bonferroni’s multiple comparison tests.

Cardiac function in CLP Tg mice was markedly improved in comparison with that in CLP WT mice based on the results from echocardiography. The ejection fraction (EF) and fractional shortening (FS) index from CLP Tg mice were significantly greater (EF, Tg 73.4 ±1.4% *vs*. WT 62.4 ±1.0%; FS, Tg40.9 ±1.4% *vs*. WT 32.0 ±1.1%) than those from CLP WT mice (*p*< 0.01; Fig 4A–4C).

**Fig 5:**
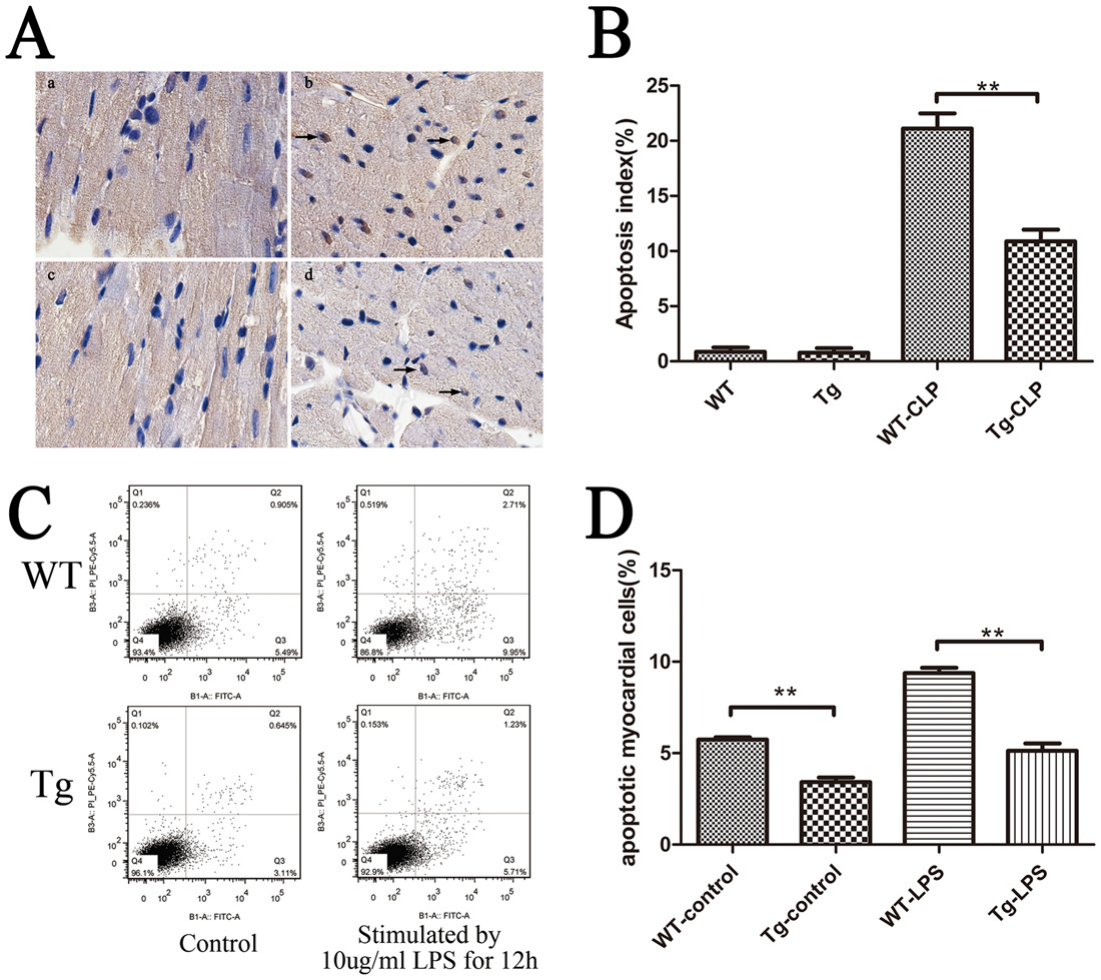
Apoptosis in the heart from WT and Tg mice. (A) Arrowheads indicate TUNEL-positive cardiomyocytes. WT (a), WT—CLP 24h (b), Tg (c), Tg—CLP 24h (d). (B)The numbers of TUNEL-positive cells in Tg mice were significantly lower than those in WT mice (Tg 10.9±2.1% *vs*. WT 21.1±3.0%) (n = 5).(C-D) The number of apoptotic cells in Tg mice were significantly lower than those in WT mice analyzed by flow cytometry (Tg 5.1±0.7% *vs*. WT 9.4±0.5%) (n = 6). Figures are representative of three independent experiments. Values represent the mean ± SEM, *p< 0.05, **p< 0.01,one-way analysis of variance (ANOVA) and Bonferroni’s multiple comparison tests.

Increased troponin levels,sensitive and specific biomarkers for cardiac injury, are predictive for the poor outcomes of septic patients [12]. Our results revealed that the levels of troponin in serum were significantly increased in both WT mice and Tg mice with sepsis. But the serum cardiac troponin I level in Tg mice increased to a significantly less degree in comparison to WT mice at 12h and 24h (*p*< 0.01; Fig 4D). To detect whether overexpression of IκBβ* can inhibit cardiomyocyte apoptosis, the apoptotic cardiomyocytes were detected by TUNEL staining at 24h post CLP. The numbers of TUNEL-positive cells in Tg mice were significantly lower than those in WT mice (Tg 10.9±2.1% *vs*. WT 21.1±3.0%) (*p*< 0.01; Fig 5A and 5B). These results were corroborated in cardiomyocyte cells treated with LPS(0 or 10μg/ml) for 12 hours *in vitro*, where the number of apoptotic cells in Tg mice were significantly lower than those in WT mice analyzed by flow cytometry (Tg 5.1±0.7% *vs*. WT 9.4±0.5%) (*p*< 0.01; Fig 5C and 5D).

### Overexpression of IκBβ* promotes cardiac autophagy

Autophagy is a process that degrades and recycles the cellular components in lysosomes. It can be induced by various stress conditions [13]. The enhanced autophagy in cardiomyocytes protects from cell injury in sepsis [14,15].

**Fig 6:**
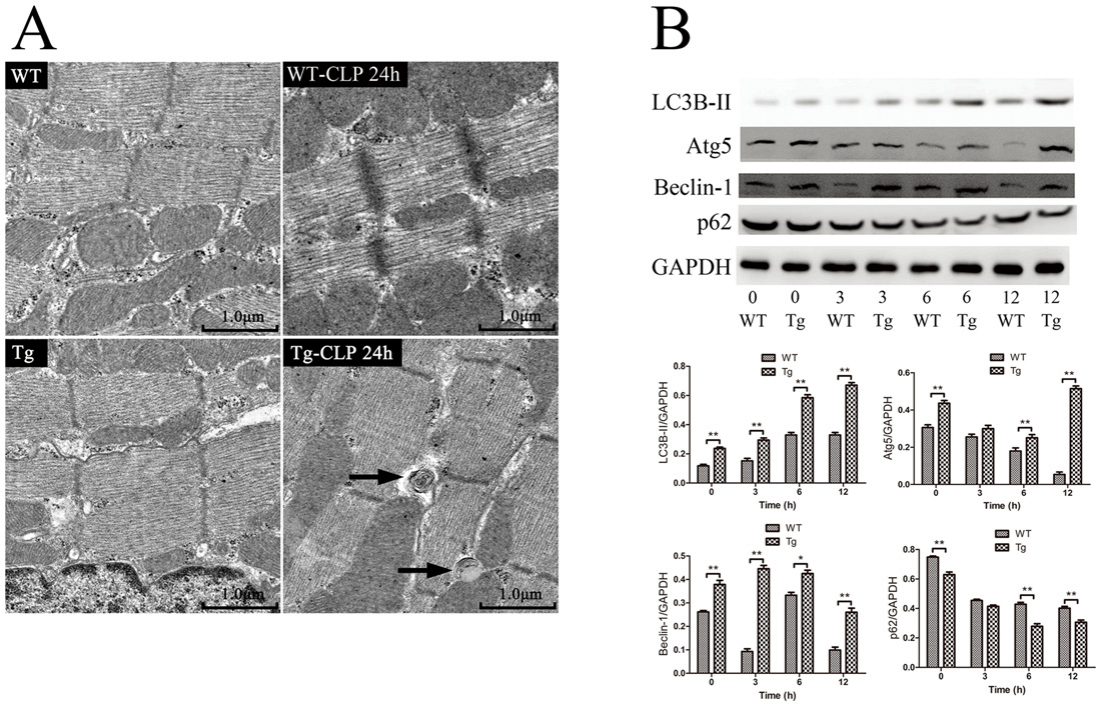
Autophagy in the heart from WT and Tg mice. (A) Electron microscopic analysis of the left ventricular myocardium from WT, WT—CLP 24h, Tg, Tg—CLP 24h mice (*n* = 3). Arrowheads indicate autophagosome. (B) The changes in autophagy related proteins. The content of autophagy proteins LC3B-II, Atg5, Beclin-1, and p62 was detected with Western Blotting. Relative protein levels were quantified and normalized to GADPH (*n* = 3). Figures are representative of three independent experiments. Values were presented as mean ± SEM, *p< 0.05, **p< 0.01, one-way analysis of variance (ANOVA) and Bonferroni’s multiple comparison tests.

To measure cardiac autophagy in sepsis, we first detected the formation of autophagicvacuoles with electron microscopy, which is the gold standard for assessing autophagy in tissue. There was a significant increase in number of autophagosomes in the heart in septic WT and septic Tg mice (1.3±0.3 *vs*. 5±0.6 per ten images; *p*< 0.01). The representative images are shown (Fig 6A).

Autophagy is classified byphagophore, autophagosome maturation, autolysosome formation, and finally degradation of target molecules. Autophagy is under the control of genes such as LC3, Atg5, Beclin-1, and p62. LC3B-II is another mediator in autophagy [16], and functions at an early step of phagophore expansion [17]. Atg5 and Beclin1 also participate in autophagosome formation. We found significant differential expression of LC3B-II, Atg5, and Beclin-1 between WT and Tg mice with or without sepsis. The upregulation of LC3B-II, Atg5, and Beclin-1 proteins in the Tg mice without sepsis suggests that IκBβ* affects the expression of Autophagy Related genes (Atgs). Moreover, the increased expression of LCB3-II,Atg5, and Beclin-1 in the heart from septic Tg mice in comparison to that from septic WT mice further demonstrates the activation of autophagy (*p*< 0.01; Fig 6B). Since p62/SQSMT1 has an adaptor function to recognize the ubiquitinylated proteins and to be removed from the cytoplasm duringautophagy, its amount is generally considered to inverselycorrelate with autophagic activity [18]. As shown in Fig 6B, the expression of p62 in the heart from Tg mice is lower than that from WT mice at 6h and 12h after CLP.

### IκBβ* mediates the enhanced expression of cathepsin L (Ctsl) in septic Tg mice

**Fig 7:**
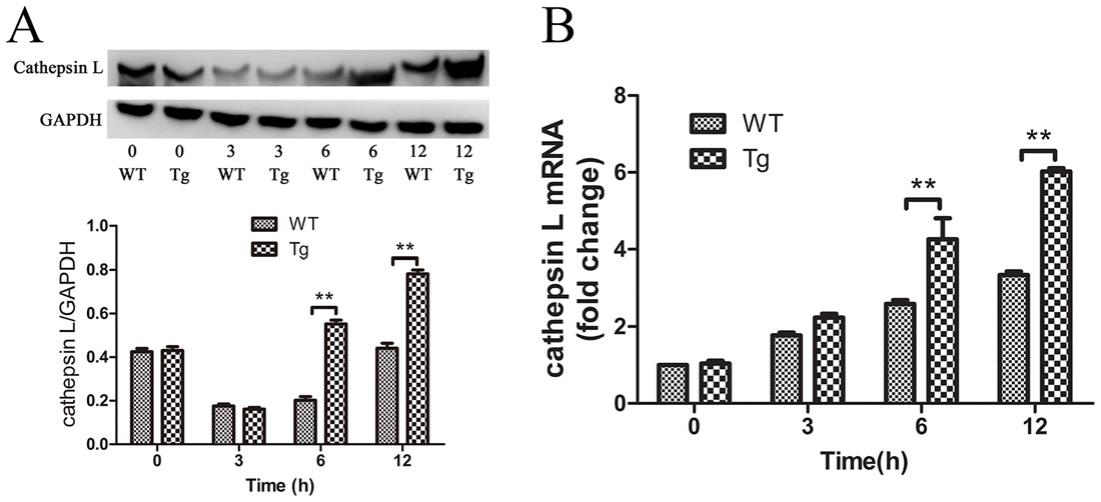
Cathepsin L expression in the heart from WT and Tg mice. (A) The expression of cathepsin L protein was detected with western blot. Relative protein levels were quantified and normalized to GADPH (*n* = 3). (B) The expression of cathepsin L at transcriptional level was examined by real-time PCR (*n* = 3). Figures are representative of three independent experiments. Values were presented as mean ± SEM, *p< 0.05, **p< 0.01, one-way analysis of variance (ANOVA) and Bonferroni’s multiple comparison tests.

Ctsl is a specific marker for mitochondrial stress response that associates with IκBβ-dependent NF-κB activation [19]. The overexpression of IκBβ* was insufficient for inducing Ctsl expression in Tg mice without sepsis. However, Ctsl expression in the septic Tg mice was significantly increased at the transcriptional and translational level compared with septic WT mice (*p*< 0.01; Fig 7).

## Discussion

The response of IκBβ to sepsis is different from other forms of NF-κB inhibitor proteins. The lower level of IκBβ protein was observed at 3 hours and the newly synthesized IκBβ gradually accumulated at 6 hours in the heart after sepsis. The degradation of IκBβ protein in the heart was also observed in an LPS model [3]. Most re-synthesized IκBβ were hypo-phospho-IκBβ-S313 in the cardiomyocytes from WT mice at 6h, and the portion of phosphorylated IκBβ increased to 73.9% at 24h (Fig 1). The phosphorylation of IκBβ-S313 by protein kinasecasein kinase II(CK2) and de-phosphorylation of IκBβ-S313 by protein phosphatase calcineurin are two important processes in controlling the phosphorylation state of IκBβ-S313. Therefore, the phosphorylation state of IκBβ at ser313 is dependent upon the balance between the protein kinase CK2 and calcineurin activities which can be enhanced by LPS [20–22].

Using IκBβ knockout mice, Wright *et al*. demonstrated mice lacking IκBβ are resistant to LPS-induced injury. Based on this result, we should expect over-expressing IκBβ to aggravate organ damage in sepsis. However, the over-expression of IκBβ could not prevent LPS-induced death and diminish serum TNF-α levels in IκBβ knock-in mice (AKBI), in which the IκBα gene is replaced with the IκBβ cDNA [23]. These paradoxical results suggest a complex role of IκBβ in the development of sepsis. The hypo-phosphorylated form of IκBβ at S313 may be involved in the process. We found the increased portion of IκBβ* in the cells and NF-κB (p65,c-Rel)in the nucleus from the heart of Tg mice(Fig 2), which may partially explain the activation of NF-κB in Tg mice. In the other words, our results in Tg mice is similar to the previous reports about the effects of hypo-phosphorylated form of IκBβ at S313 on NF-κB [24]. Besides IκBβ* facilitates the nuclear translocation and activation of NF-κB, persistent degradation of IκBβ may also lead to activation of NF-κB. Therefore it is an interesting topic to study the synthesis, degradation and location of NF-κB/IκBβin the nucleus and cytoplasm in Tg mice with or without sepsis in the future [6].

WT IκBβ binds with subunits of NF-κB in the cytoplasm and prevents translocation of p65, p50, c-Rel into nucleus. Unlike the WT IκBβ, the hypo-phosphorylated IκBβ at S313 and p65:p50 could form ternary complexes to transport to the nucleus, in which it fails to block NF-κB DNA binding activity and NF-κB directed transcription. What is even more important is that the complexes are resistant to dissociation by IκBα. All these factors increase NF-κB activation [5,25,26].

To examinethe pathological significance of the hypo-phosphorylated form of IκBβ at S313 in sepsis, we usedTg mice that over-express mutated IκBβ at S313 in the present experiment. Surprisingly, the Tg mice are resistant to sepsis and have lower levels of proinflammatory cytokines in comparison with WT mice (Fig 3). Recently, Rao *et al*. reported that deficiency of IκBβ in knockout mice blocks the formation of p65:c-Rel and hypo-phosphorylated IκBβ complexes, which bind to TNF-α or IL-1β promoter and increase transcription after LPS stimulation [24,27]. Unlike the expectation that overexpression of hypo-phosphorylated IκBβ would result in pro-inflammatory cytokine production, we observed a decrease of TNF-α and IL-6 at the transcriptional level in the heart and also a reduction in protein concentration in serum in septic Tg mice(Fig 3). This discrepancy may be explained by the enhanced autophagy that diminishes inflammatory mediators including IL-6, TNF-α,IL-10, and MCP-1 [28].

The activation of NF-κB plays important roles in heart damage following sepsis. Theprotective effects of IκBβ* in heart injury was revealed by improvedcardiac function and decreased serum troponin I (Fig 4) in CLP Tg mice in comparison with those in CLP WT mice. We demonstrated that IκBβ* can inhibit the occurrence of apoptosis in sepsis (Fig 5). This result concurs with a report in a mitochondrial stress model [17]. Biswas *et al*. found that mutations at S313/S315 of human IκBβ is essential for initiating the mitochondrial stress response and facilitating resistance to apoptosis [5,17]. However, the IκBβ overexpression alone did not prevent oxidant stress—induced apoptosis [29]. From this evidence, we speculate the anti-apoptosis effect of IκBβ* depends on the mutation site of IκBβ at Ser313. Recently, Tang *et al*. reported LPS induced NF-κB activation through IκBβ degradation induces apoptosis in fetal pulmonary arterial endothelial cells. Unfortunately, they did not analyze the role of hypo-phosphorylated IκBβ at Ser-313 [30]. Besides inhibiting apoptosis, IκBβ* overexpression also induces autophagy in the heart following sepsis as shown in Fig 6. The increased autophagy in Tg mice may result from activation of NF-κB, which subsequently mediates changes in autophagy related proteins including LC-3, Beclin-1, Atg5, and p62 [31,32]. Over-expression of hypo-phosphorylated IκBβ at Ser-313 can increase the translocation of cytoplasmic NF-κB p65 into nucleus, in which it binds to the promoter of Beclin-1 and LC-3B, leading to their transcriptional activation [33,34]. In a model of heat shock, autophagy is drastically decreased in p65-depleted cells [35,36]. These results suggest the roles of NF-κB in regulating autophagy.

However, the conflicting data show that the activation of NF-κB represses autophagy by mTOR in TNF-αtreated Ewing sarcoma cells. A possible reason for the discrepancy might be stimulation or repression of autophagy by different subunits of NF-κB [37,38].

In addition to activation of NF-κB, IκBβ* at Ser313 can mediate the mitochondrial stress response in which Ctsl is a specific marker. Our results further confirmed the roles of IκBβ* in upregulation of Ctsl in Tg mice post sepsis (Fig 7). Ctsl is a lysosomal cysteine endopeptidase that plays important roles in degradation of auto-phagolysosomal content as evidenced in knockout mice [39]. Since degradation of auto-phagolysosomal content is impaired in cardiomyocytes in sepsis, Ctsl may help to complete the autophagic process and exert a protective effect on the heart in septic Tg mice [28,40]. However, the roles of mitochondria stress in protecting against heart injury in Tg mice are not fully understood. Because hypo-phosphorylated form of IκBβ at S313 is a natural form in sepsis and it has many beneficial effects on heart injury after sepsis, we may treat the disease through increasing the protein level or developing a specific peptide to mimic the beneficial effects of this protein.

## Conclusions

We demonstrated the beneficial effects of IκBβ* on heart injury in sepsis through diminishing apoptosis andimprovingautophagy.

## Supporting Information

S1 Dataset The primary data for IκBβ expression in the heart from WT mice post CLP. (XLSX)

S2 Dataset The primary data for phenotype of IκBβ in WT and Tg mice. (XLSX)

S3 Dataset The primary data for expression of EGFP in Tg mice post CLP, survival rate and expressionof inflammatory cytokines in WT and Tg mice post CLP. (XLSX)

S4 Dataset The primary data for cardiac function in WT and Tg mice. (XLSX)

S5 Dataset The primary data for apoptosis in the heart from WT and Tg mice. (XLSX)

S6 Dataset The primary data for autophagy in the heart from WT and Tg mice. (XLSX)

S7 Dataset The primary data for cathepsin L expression in the heart from WT and Tg mice. (XLSX)
